# *Wickerhamomyces anomalus* Pseudo-Outbreak Associated with Medical Lubricating Gel, South Africa, 2023–2024

**DOI:** 10.3201/eid3209.260608

**Published:** 2026-09

**Authors:** Caroline Maluleka, Husna Ismail, Liliwe Shuping, Ruth Mpembe, Gift Sandhleni, Serisha Naicker, Tsidiso Maphanga, Nelesh P. Govender

**Affiliations:** National Institute for Communicable Diseases, Johannesburg, South Africa (C. Maluleka, H. Ismail, L. Shuping, R. Mpembe, G. Sandhleni, S. Naicker, T. Maphanga, N.P. Govender); University of Witwatersrand School of Pathology, Johannesburg (C. Maluleka, L. Shuping, T. Maphanga, N.P. Govender)

**Keywords:** Wickerhamomyces anomalus, fungi, fungal infections, lubricating gel, pseudo-outbreak, South Africa

## Abstract

A nationwide investigation identified 426 patients with *Wickerhamomyces anomalus* fungal cultures in South Africa; most cases clustered during May–November 2023. Case-patients lacked evidence of infection, indicating a pseudo-outbreak. Genomic and laboratory analyses implicated contaminated sterile lubricating gel. A nationwide product recall resulted in a decline in reported cases.

*Wickerhamomyces anomalus*, an environmental ascomycetous yeast, is associated with healthcare-associated outbreaks among critically ill or immunocompromised patients, but its sources are rarely identified (*1*–*5*). Contaminated medical gel has been implicated in healthcare-associated outbreaks caused by water-associated bacteria (*6*–*11*). In November 2023, the National Institute for Communicable Diseases (NICD) was alerted to an unusually high frequency of *W. anomalus* isolation at a laboratory in KwaZulu-Natal Province, South Africa. Two hospitals in the Western Cape Province separately identified 2 clusters during July–August 2023. One cluster was associated with recent central venous catheter placements through which blood cultures subsequently were collected; a sterile lubricating gel (Lubri-A sterile lubricating jelly; Electro-Spyres, https://www.electrospyres.com) was used in the ultrasound-guided catheter insertion procedure and was a suspected source of pseudo-infections. We investigated the extent, source, and clinical relevance of a nationwide pseudo-outbreak.

## The Study

We reviewed National Health Laboratory Service (NHLS) records for patients at public-sector healthcare facilities with culture-confirmed *W. anomalus* from any specimen type during January 1, 2022–April 29, 2024. We removed repeat isolates from the same patient within 30 days. We contacted laboratories to determine whether increased detection coincided with introduction of the Vitek MS system (bioMérieux, https://www.biomerieux.com). We retrospectively collected clinical case data at 2 hospitals. We received *W. anomalus* isolates cultured from clinical specimens and unopened sterile lubricating gel sachets in circulation at healthcare facilities. We subcultured the isolates onto chromogenic agar (Candida CHROMagar, https://www.chromagar.com) and incubated them in ambient air at 35–37°C for 18–24 hours. We confirmed species identification by using matrix-assisted laser desorption/ionization time-of-flight mass spectrometry (MALDI BioTyper; Bruker, https://www.bruker.com). We performed antifungal susceptibility testing by using the Sensititre YeastOne method (Thermo Fisher Scientific, https://www.thermofisher.com). We interpreted MICs by using the Clinical and Laboratory Standards Institute M57S epidemiologic cutoff values (*12*).

We analyzed whole-genome sequences, generated by the NextSeq platform (Illumina, https://www.illumina.com), by using FungiPhyloGen pipeline (https://zenodo.org/records/18121445). We assessed sequence quality by using FASTQC version 0.11.9 (https://sourceforge.net/projects/fastqc.mirror/files/v0.11.9) and MultiQC version 1.13 (https://github.com/MultiQC/MultiQC/releases), followed by trimming with TrimGalore version 0.6.7 (https://github.com/FelixKrueger/TrimGalore/releases). We aligned filtered reads to a reference strain from the United States (*W. anomalus* NRRL Y-366-8) (*13*) by using BWA version 0.7.19 (https://github.com/lh3/bwa/releases). We annotated high-confidence single-nucleotide polymorphisms (SNPs) (allele frequency >0.85) and used them for distance matrix generation and phylogenetic reconstruction with IQ-TREE version 2.2.0.3 (https://iqtree.github.io) by using 1,000 bootstrap replicates to assess clade support.

**Figure 1:**
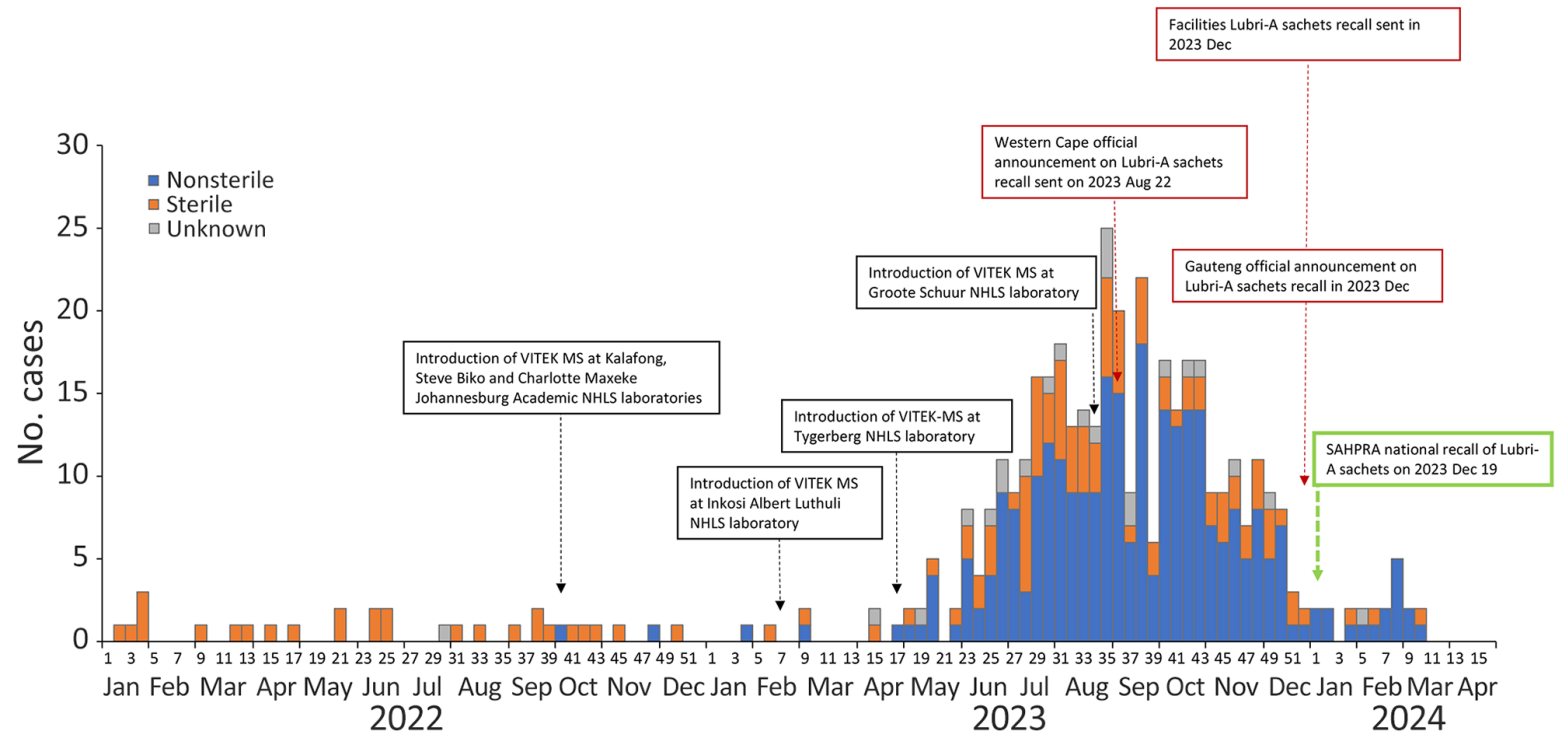
Annotated epidemic curve of *Wickerhamomyces anomalus* infection cases, by epidemiologic week, South Africa, January 1, 2022–April 29, 2024. Normally sterile-site specimens were those obtained from anatomical sites expected to be free of viable microorganisms. Nonsterile-site specimens were those obtained from sites normally colonized by microbiota or exposed to the external environment. Lubri-A refers to Lubri-A sterile lubricating jelly (Electro-Spyres, https://www.electrospyres.com), Vitek MS to the Vitek MS system (bioMérieux, https://www.biomerieux.com). NHLS, National Health Laboratory Service, SAPHRA, South African Health Products Regulatory Authority.

We identified 426 cases across 7 of 9 provinces in South Africa over the study period (Figure 1). Before April 1, 2023, the baseline was 2–3 cases per facility per month; 346/426 (81%) cases occurred during May–November 2023. The increase did not coincide with introduction of the Vitek MS system. The most frequent specimen categories were urine (207/426 [49%]) and blood (77/426 [18%]) (Appendix Figure 1). Gauteng Province had the highest number of cases (180/426 [42%]), and Western Cape Province had the highest number of cases from sterile sites (Appendix Figure 2).

Clinical information was available for 52/426 (12%) case-patients (Table). Median age was 23 years (interquartile range 0–47 years); 31/49 (63%) patients were male, and 21 (37%) female. Among 51 of the 52 patients, *W. anomalus* was recovered from urine (26 [51%]), tracheal aspirate (13 [26%]), peritoneal dialysis fluid (6 [12%]), and blood (4 [8%]). Among 37 patients with procedure information, 23 (62%) underwent urinary catheterization and 12 (32%) underwent endotracheal intubation; procedures were not mutually exclusive. For 48 patients whose samples were positive for *W. anomalus*, clinicians considered the positive culture as contamination in 20 (42%) and clinically relevant in 28 (58%) patients: urinary tract infection in 15 (31%), lower respiratory tract infection in 8 (17%), and bloodstream infection in 2 (4%). Antifungal treatment was administered to 10 (23%) of 44 patients for whom treatment information was available. However, objective evidence of infection such as signs of sepsis or elevated levels of inflammatory biomarkers was absent or incompletely documented. Only 5 patients had *W. anomalus* isolated from subsequent cultures. Among 50 patients with known outcomes, 30 (60%) were discharged, 6 (12%) remained hospitalized, and 14 (28%) died; available data were insufficient to attribute deaths to *W. anomalus* infection (Appendix Figure 2).

**Table:** Characteristics of patients with *Wickerhamomyces anomalus* infection in 2 hospitals, South Africa, August 7, 2023–January 11, 2024*

Characteristic	Value
Median age, y (IQR)	23 (0–47)
Sex, n = 49	
F	18 (37)
M	31 (63)
Underlying condition or procedure, n = 36	
HIV	6 (17)
Chronic kidney disease	7 (19)
Surgical procedure	10 (28)
Other	23 (64)
Gel-use–linked procedure, n = 38†	
Urinary catheterization	24 (63)
Endotracheal intubation	13 (48)
Ultrasound scan	10 (42)
Other‡	10 (42)
Specimen type, n = 51	
Urine	26 (51)
Tracheal aspirate	13 (26)
Peritoneal dialysis fluid	6 (12)
Blood	4 (8)
Bronchial alveolar lavage	1 (2)
Tissue	1 (2)
*W. anomalus*–linked diagnosis by treating clinician, n = 48	
Considered a contaminant	20 (42)
Urinary tract infection	15 (31)
Lower respiratory tract infection	8 (17)
Bloodstream infection	2 (4)
Peritonitis	3 (6)
Antifungal treatment administered, n = 44	
Yes	10 (23)
No	34 (77)
Adverse events related to *W. anomalus* infection, n = 51	
No	30 (59)
Unknown	21 (41)
Outcome, n = 50	
Still admitted	6 (12)
Discharged	30 (60)
Died	14 (28)

*Values are no. (%) except as indicated; IQR, interquartile range. †Denominators vary because of missing data; some persons had >1 procedure linked to the use of lubricating gel. ‡Includes nasogastric tubing (n = 5), bronchoscopy (n = 1), rectal enema (n = 1), Pap smear (n = 1), vaginal examination and cardiotocography (n = 1), and tracheal dilation (n = 1).

Of 86 isolates, 62 (72%) were cultured from clinical specimens and 24 (28%) from gel batches. Sealed gel sachets uniformly yielded heavy fungal growth. We confirmed 85 (99%) isolates as *W. anomalus*; we excluded 1/86 (1%) *Candida albicans* isolate. Seventeen clinical isolates and 11 gel isolates underwent susceptibility testing. Fluconazole MIC_50_ (MIC at which 50% of a specific isolate population is inhibited from growth) was 2 μg/mL for clinical and gel isolates. Fluconazole MICs were <2 μg/mL for 16/17 (94%) clinical isolates and 9/11 (82%) gel isolates; all were wild-type. All other azole and micafungin MICs were at or below their respective epidemiologic cutoff values. Amphotericin B MICs were <1 μg/mL for 15/17 (88%) clinical isolates and 9/11 (82%) gel isolates; 2/17 (12%) clinical isolates and 2/11 (18%) gel isolates had MICs >1 μg/mL. Flucytosine MICs were 0.06 μg/mL for all 17 clinical isolates, whereas 9/11 (82%) gel isolates had MICs >64 μg/mL (Appendix Table 1).

**Figure 2:**
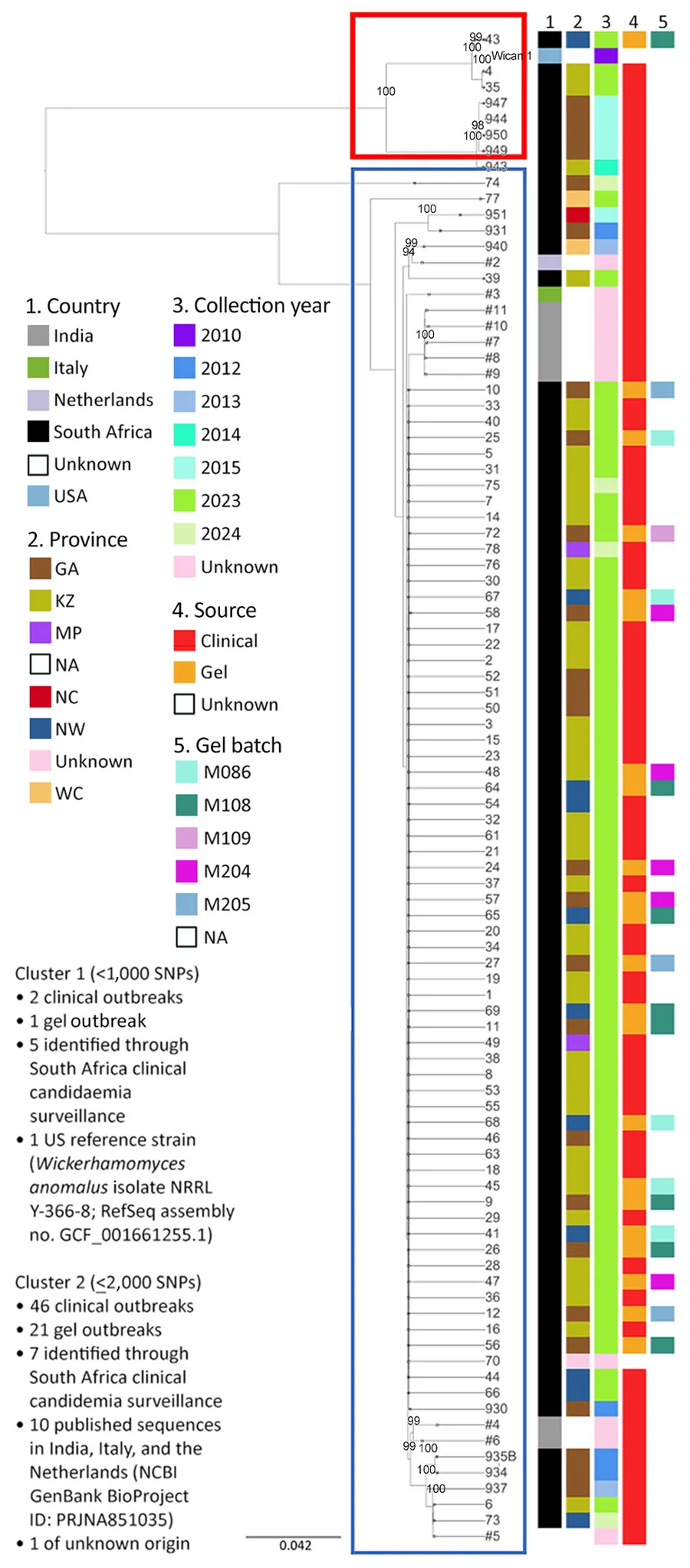
Whole-genome SNP phylogeny of *Wickerhamomyces anomalus* clinical, gel, surveillance, and reference isolates, South Africa. Red box indicates cluster 1; blue box indicates cluster 2. Scale bar indicates mean number of nucleotide substitutions per variable site. Node values represent ultrafast bootstrap support percentages based on 1,000 replicates. GA, Gauteng Province; KZ, KwaZulu–Natal Province; MP, Mpumalanga Province; NA, not applicable; NC, Northern Cape Province; NCBI, National Center for Biotechnology Information; NW, North West Province; WC, Western Cape Province; SNP, single-nucleotide polymorphism; USA, United States.

The phylogenetic dataset included 95 genome sequences. Of those, 72 (76%) were current outbreak isolates (GenBank BioProject no. PRJNA148538): 49 (68%) clinical, 22 (31%) gel-derived, and 1 (1%) undocumented source. The remaining 23 included 12 historic surveillance isolates from South Africa, 10 published genomes (BioProject no. PRJNA851035), and 1 reference genome (*W. anomalus* NRRL Y-366-8; RefSeq [https://www.ncbi.nlm.nih.gov/refseq] no. GCF_001661255.1). Two major clusters were separated by >10,000 SNPs; clinical and gel-derived genomes were interspersed within both clusters (Figure 2). Within-cluster diversity was <1,000 SNPs in the smaller cluster and <2,000 in the larger cluster. That finding indicated a contaminated product rather than a single local transmission chain.

The outbreak was reported to the South African Health Products Regulatory Authority and the National Department of Health on August 21, 2023. On December 8, 2023, the National Department of Health instructed facilities to stop using implicated batches, quarantine them, halt distribution, obtain alternative products, and establish case-based surveillance.

After an independent investigation, the South African Health Products Regulatory Authority later issued a Class 1 Type A nationwide recall and informed other regulatory authorities. Case counts declined sharply thereafter, although sporadic detection persisted in early 2024, probably because of remaining facility stock still in use (Figure 1).

## Conclusions

Epidemiologic, microbiologic, and genomic findings identified single-use sterile lubricating gel sachets contaminated at manufacture as a source of a national pseudo-outbreak of *W. anomalus* infection. Evidence included detection across 7 provinces, recovery of the organism from sealed sachets and 3 separate batches, interspersion of clinical and gel genomes, absence of a temporal association with laboratory instrument changes, and a rapid case decline after the nationwide recall. Many positive cultures probably reflected contamination of specimens or devices rather than infection. Nonetheless, treating clinicians considered the organism clinically relevant in more than half of patients in the clinical subset, and 23% received antifungal treatment. Those findings illustrate how pseudo-outbreaks can lead to potentially unnecessary treatment. Interpretation was limited because clinical data were only available for 12% of the cases from 2 hospitals and collected retrospectively. 

Healthcare facilities and diagnostic laboratories should be suspicious when unusual organisms are repeatedly detected across multiple specimen types or in geographically separate facilities. Products labeled sterile remain a risk if assurance of sterility fails during manufacturing. Strengthened procurement, supplier quality assurance, product traceability, postmarket surveillance, notification, and rapid national coordination among laboratories, clinicians, infection prevention teams, public health authorities, and regulators are essential to prevent and contain similar events.

Appendix Additional information about *Wickerhamomyces anomalus* pseudo-outbreak associated with medical lubricating gel, South Africa, 2023–2024.
