# Supplementary material for: Wickerhamomyces anomalus Pseudo-Outbreak Associated with Medical Lubricating Gel, South Africa, 2023–2024
Source: Emerg Infect Dis. 2026 Sep;32(9):1492–6. doi: 10.3201/eid3209.260608 (PMC13555721; doi:10.3201/eid3209.260608)
Supplement: Supplementary file 1 [file 26-0608-Techapp-s1.pdf]

Article DOI: https://doi.org/10.3201/eid3209.260608

EID cannot ensure accessibility for supplementary materials supplied by authors. Readers who have difficulty accessing supplementary content should contact the authors for assistance.

Wickerhamomyces anomalus Pseudo-Outbreak Associated with Medical Lubricating Gel, South Africa, 2023–2024

Appendix

Appendix Table 1. Minimum inhibitory concentrations of clinical and lubricating-gel *Wickerhamomyces anomalus* isolates, South Africa.

Antifungal agent (ECV µg/mL)	Clinical isolates, no. tested	Clinical MIC_{50}	Clinical MIC_{90}	Gel isolates, no. tested	Gel MIC_{50}	Gel MIC_{90}
Fluconazole (8)	17	2	2	11	2	4
Voriconazole (0.25)	17	0.06	0.12	10	0.09	0.12
Itraconazole (1)	17	0.12	0.25	11	0.12	0.25
Posaconazole (2)	17	0.25	0.5	11	0.5	1
Anidulafungin (N/A)	17	0.015	0.016	11	0.015	0.015
Micafungin (0.12)	17	0.03	0.03	11	0.03	0.03
Caspofungin (N/A)	17	0.03	0.06	11	0.03	0.06
Flucytosine (N/A)	17	0.06	0.06	11	64	64
Amphotericin B (1*)	17	0.5	2	11	0.5	2

Abbreviations: ECV, epidemiologic cut-off value; MIC, minimum inhibitory concentration; N/A, no published ECV.
Note: Values are µg/mL. ECVs are not clinical breakpoints.
*Amphotericin B MICs were determined by E-test; all other MICs were determined by Sensititre Yeast One broth microdilution.

Appendix Table 2: Clinical characteristics of cases of *Wickerhamomyces anomalus* who died while admitted at two hospitals, August 7, 2023 through January 11, 2024 (n=11).

Sex	Age	Ward type	Diagnosis	Specimen type	*W. anomalus*–related diagnosis	Antifungal treatment for *W. anomalus*
M	40y	ICU	Olfactory groove meningioma	ETA	Contaminant	No
M	9d	ICU	DiGeorge syndrome	CU	Contaminant	No
M	48y	ICU	Alcohol induced pancreatitis, multi-organ failure, hypertension	ETA	LRTI	Yes
M	5m	ICU	Acyanotic heart disease, dilated cardiomyopathy, hypertrophic cardiomyopathy, *Candida auris* sepsis	ETA	Contaminant	No
M	43y	Unknown	Unknown	Urine	Unknown	Unknown
M	10d	NICU	Meningitis, neonatal sepsis, necrotizing enterocolitis	BC	BSI	No
M	61y	Unspecified	Suspected UTI with acute kidney injury, cerebrovascular insufficiency	Urine	UTI	No
M	84y	Unspecified	Acute decompensated heart failure, grade II acute kidney injury, sepsis	Urine	UTI	No
M	85y	Medical/Oncology	Prostate cancer, bilateral hydrocele, hypernatremia, dyslipidemia, dementia, UTI, hypertension	Urine	UTI	No
F	53y	Oncology	Metastatic adenocarcinomas, acute kidney disease, ovarian cancer	Urine	Contaminant	No
M	82y	ICU	Chronic obstructive pulmonary disease	Urine	UTI	Unknown
M	2m	ICU	Hydrocephalus, brainstem dysfunction	Urine	Contaminant	No
F	1m	NICU	Gastroschisis, intrauterine growth restriction	BC	Bloodstream infection	Yes
F	1m	NICU	Gastroschisis	ETA	Unknown	Yes

ICU: Intensive care unit; NICU: Neonatal ICU; ETA: Endotracheal tube aspirate; CU: Catheter urine; BC: Blood culture; BSI: Bloodstream infection; UTI: Urinary tract infection; LRTI: Lower respiratory tract infection

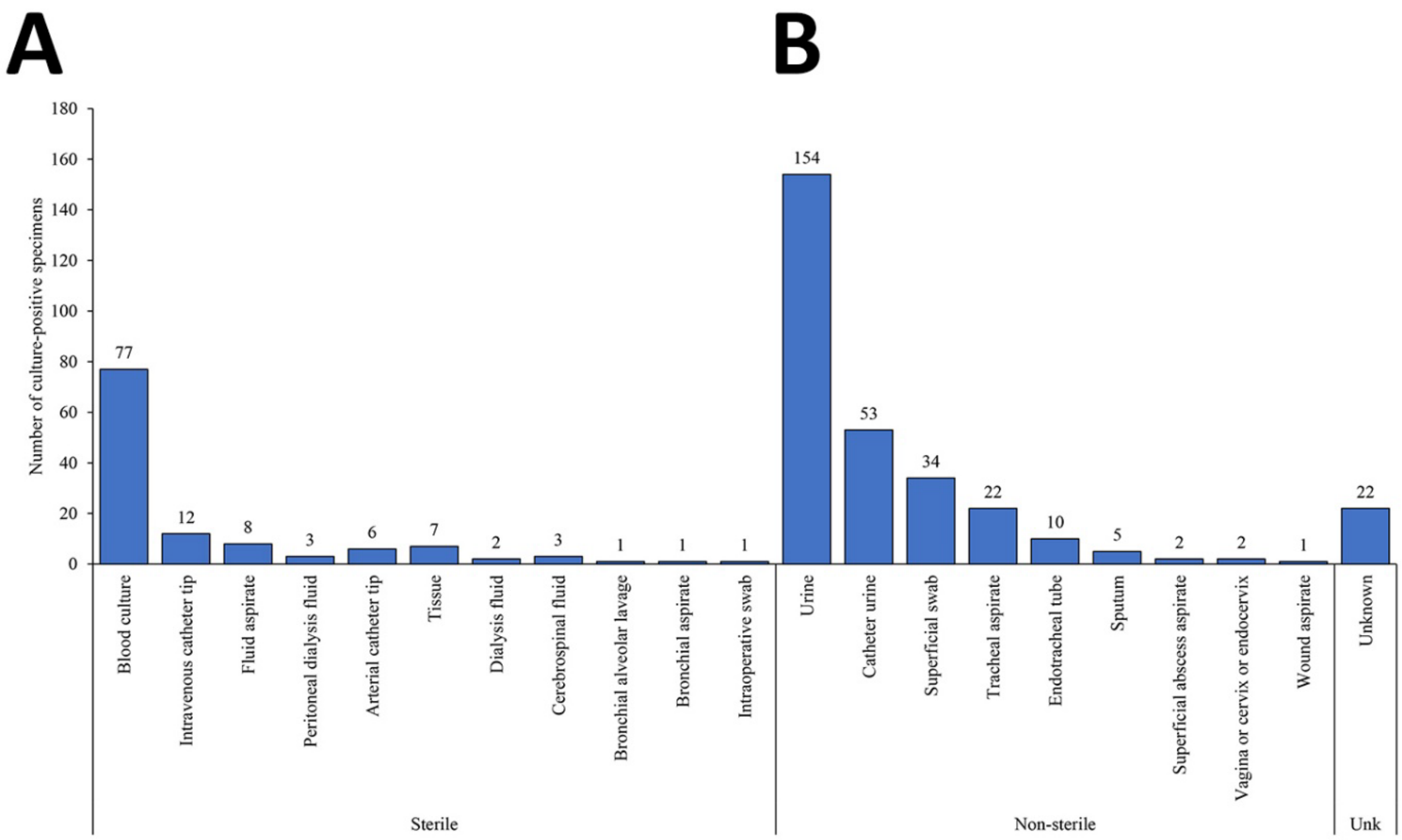

Appendix Figure 1. Number of culture-positive *Wickerhamomyces anomalus* cases by specimen type, South Africa, January 1, 2022–April 29, 2024. Normally sterile-site specimens were those obtained from anatomical sites expected to be free of viable microorganisms. Nonsterile-site specimens were those obtained from sites normally colonized by microbiota or exposed to the external environment.

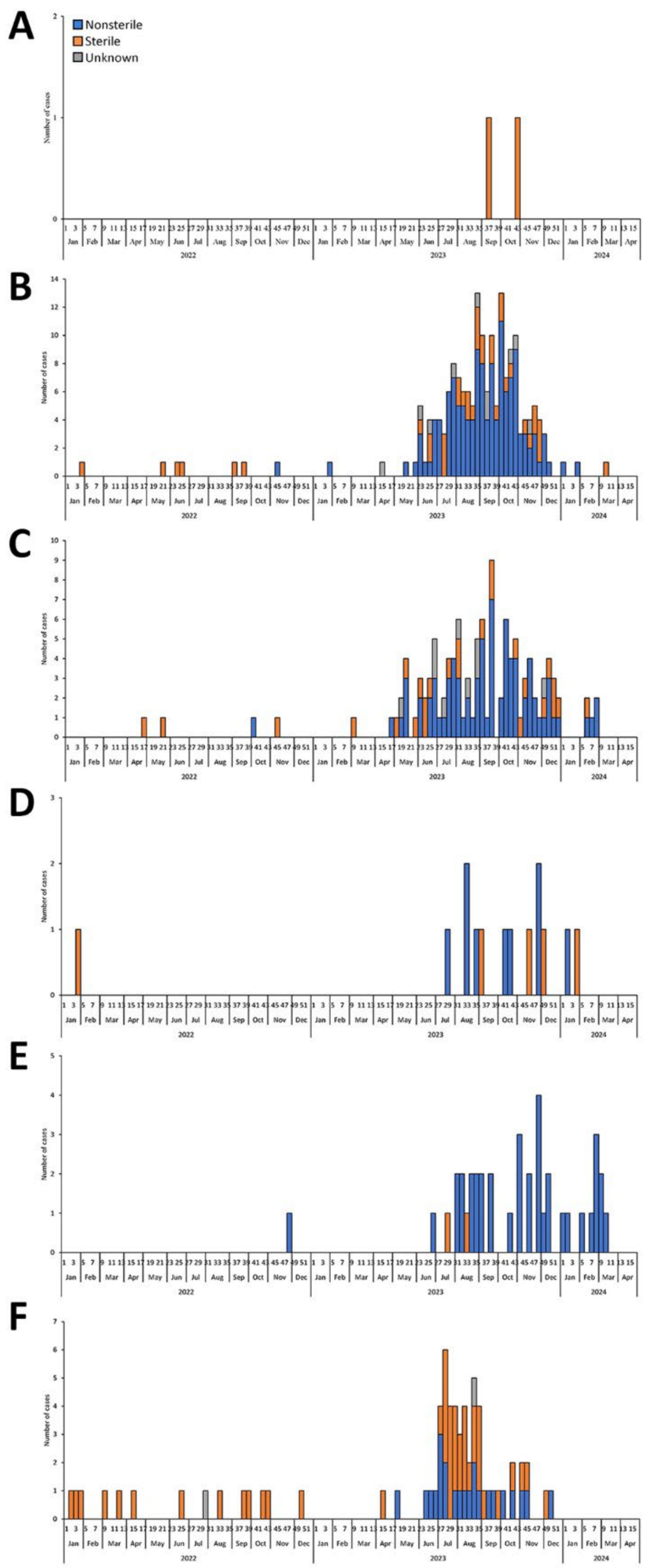

Appendix Figure 2. *Wickerhamomyces anomalus* cases from sterile and nonsterile specimens by epidemiologic week and province, South Africa, January 1, 2022–April 29, 2024. A) Free State, n = 2; B) Gauteng, n = 180; C) KwaZulu–Natal, n = 115; D) Mpumalanga, n = 14; E) North West, n = 39; F) Western Cape, n = 68.
